# Knowledge and Practices Regarding Human Papillomavirus and Cervical Cancer Screening Among Women in Low-Income Areas of China: A Cross-Sectional Study

**DOI:** 10.7759/cureus.55930

**Published:** 2024-03-11

**Authors:** Jiaojiao Chen, Ruoyi Zhang, Wei Xu, Li Bai, Dehua Hu, Yuxian Nie, Rumei Xiang, Dan Kang, Qiu-ling Shi

**Affiliations:** 1 College of Public Health, Chongqing Medical University, Chongqing, CHN; 2 Obstetrics and Gynecology, Centre of Maternal and Child Health, Shaanxi, CHN; 3 State Key Laboratory of Ultrasound in Medicine and Engineering, Chongqing Medical University, Chongqing, CHN; 4 College of Public Health, State Key Laboratory of Ultrasound in Medicine and Engineering, Chongqing Medical University, Chongqing, CHN

**Keywords:** awareness, cross-sectional, screening, cervical cancer, human papillomavirus

## Abstract

Background: Persistent human papillomavirus (HPV) infection is the primary cause of cervical cancer. However, this can be prevented through vaccination and screening. This study aimed to clarify the relationship between behavior, knowledge, and attitude toward cervical cancer and regular screening and HPV infection among women in Lueyang County.

Methods: Women who underwent cervical cancer screening at the outpatient department of a maternal and child health center between September and December 2021 were invited to participate. In total, 2,303 women completed the questionnaire. Women who underwent regular or irregular screening were 1:1 matched for age. Differences in knowledge of HPV and attitudes toward HPV vaccination among different populations were assessed. Logistic regression analysis was performed to identify the factors influencing HPV infection.

Results: In total, 417 pairs of women who underwent regular and irregular screening were successfully matched. Multivariate logistic regression results indicated that age is a risk factor for HPV infection (OR=1.056 95%CI: [1.031 1.082]), while regular screening acts as a protective factor against HPV infection (OR=0.174 95%CI: [0.117 0.259]). Additionally, regular screening was associated with a higher level of knowledge about HPV among women compared to those who did not undergo regular screening (p<0.001).

Conclusions: Women in Lueyang County have low levels of knowledge regarding HPV and cervical cancer. Regular screening is a protective factor against HPV infection. The regular screening group demonstrates a higher level of HPV knowledge compared with the irregular screening group. These findings highlight the importance of regular screening and the need to strengthen public health education.

## Introduction

Cervical cancer is the only cancer with a definitive cause attributed to persistent infection by high-risk human papillomavirus (HPV) [1]. Therefore, further exploration in this field is warranted in future research to bolster HPV vaccination, regular screening, and other efficacious measures aimed at reducing the incidence and mortality of cervical cancer, as evidenced in developed nations [2,3]. Thus, in 2009, under the auspices of the All-China Women’s Federation and the Ministry of Health, China launched a screening program for “two cancers” (cervical and breast cancers) and implemented free cervical cancer screening for rural women aged 35-64 years [4]. However, despite the widespread recognition of cervical cancer screening, it has low participation rates, especially in rural and western regions, compared with eastern regions. The disparity in participation rates across various socioeconomic and geographic provinces poses a significant challenge in preventing cervical cancer [5]. Currently, China’s cervical cancer screening rate is far below the target of the World Health Organization of 70% screening coverage [6].

Multiple elements impact women's engagement in cervical cancer screening, encompassing age, educational attainment, socioeconomic standing, and awareness of cervical cancer [7]. Previous studies have demonstrated a significant correlation between cervical cancer knowledge and the rate of screening participation [8,9]. An insufficient understanding of cervical cancer is considered one of the most important factors affecting participation rates [10]. That is, women who have participated in screening generally have greater knowledge of cervical cancer than those who have not undergone screening [11].

Lueyang County, which has high cervical cancer incidence and mortality rates, is a low-income region in China [12]. With an average educational level below the national standard, most of the residents live as farmers or migrant workers. Despite the initiation of annual cancer screenings in 2017, orchestrated by the local maternal and child health centers in collaboration with regional health centers and village doctors, the uptake in Lueyang County remains low. A limited number of women benefit from free screening, whereas several abstain from participation, which is potentially attributable to insufficient health awareness, financial constraints, and logistic complications [13].

This study aimed to examine the correlation between cervical cancer knowledge and adherence to regular screening practices. Additionally, we aimed to evaluate the effectiveness of regular screening for early detection of cervical cancer. Moreover, the present study aimed to understand women’s attitudes toward cervical cancer prevention in high-risk areas. The primary objective was to increase the adoption of regular cervical cancer screening by developing evidence-based health education strategies aimed at enhancing understanding of cervical cancer and, consequently, its prevention.

## Materials and methods

Study design and ethical considerations

This study used a cross-sectional design and was conducted at Lueyang County Maternal and Child Health Hospital in Hanzhong City, Shaanxi Province. We collected information from women who underwent cervical cancer screening and invited them to complete our questionnaire. The content of our questionnaire was derived from a literature review and expert consultation. After obtaining informed consent from the participants, professional nurses provided guidance on completing the questionnaire. This study was approved by the Ethics Committee of Lueyang County Maternal and Child Health Hospital (Approval number: 2021-001).

Data collection and inclusion criteria

We invited all women who underwent cervical cancer screening from September to December 2021 to complete a paper-based questionnaire, and the completed questionnaires were subsequently entered into a data platform by professional personnel. Additionally, we registered the HPV test results of all patients on the data platform. Women from Lueyang County aged 20 or more and agreed to participate in the survey were included. Women with mental illnesses, depression, and a history of cervical cancer were excluded. Ultimately, 2,303 women participated in our study.

Questionnaire

The questionnaire included demographic information, health-related knowledge about HPV, regular participation in cervical cancer screening, and attitudes toward HPV vaccination. We defined women who had been screened for cervical cancer in the last 3 years as regularly screened and women who had not been screened for cervical cancer in the last 3 years as not regularly screened.

Notably, there are a total of seven questions of HPV health-related knowledge, the first question is “Do you know about HPV?”, Only if the answer is “YES” can you continue to answer the next six questions. The next six questions are “HPV is an important etiological factor of cervical cancer” “HPV can cause genital warts in both men and women” and “HPV can be transmitted through sexual contact” “Most HPV infections can naturally regress without treatment” “Men can also be infected with HPV” “Condoms can prevent HPV infection” Participants answered “Know” or “Unknown” based on their perceptions.

Attitude refers to the willingness to receive the HPV vaccine, with a positive attitude indicating willingness to receive the HPV vaccine and a negative attitude indicating unwillingness to receive the HPV vaccine.

Although no formal validation of this questionnaire was performed, we performed quality controls, professional nurses training, and extensive reviews throughout the entire study. This included standardized training for all surveyors prior to the commencement of the official survey to ensure that they fully understood the research content and questionnaire items and to unify operational standards. During the survey, surveyors checked the completeness of the questionnaire responses and reminded participants to fill in any missing items. Post-survey, the validity of the questionnaires was reviewed to exclude invalid responses, and finally, data from the questionnaires were entered using a dual-entry system.

Statistical analysis

For descriptive analysis, SAS version 9.4 was employed, while SPSS was utilized for participant matching. Participant demographic and clinical characteristics were summarized, with continuous variables presented as mean±standard deviation and categorical variables as proportions (%). Significant associations in contingency tables were tested using standard Pearson’s chi-squared test. If the P-value calculated in these analyses was <0.05, the difference was considered significant. We aimed to analyze the differences in HPV knowledge between the regular and irregular screening groups. To increase the effects of reducing confounding factors and enhance the comparability of the regular and irregular screening groups, we established the regular and irregular screening groups using age as a matching variable, with 1:1 matching. Additionally, multivariate logistic regression was used to assess the influencing factors of HPV infection.

This article was previously posted to the medRxiv preprint server on November 16, 2023.

## Results

In total, 2,303 women participated in this study, 419 (mean age, 47.54±8.48 years) in the regular screening group and 1,884 (mean age, 49.26±8.45 years) in the irregular screening group. Out of the participants, a total of 465 participants tested positive for HPV. A total of 39.67% and 51.98% of women in the regular and irregular screening groups, respectively, were farmers. In the regular and irregular screening groups, 195 (47.33%) and 816 (43.54%) women, respectively, had secondary education (middle/high school). Furthermore, a greater proportion of women in the regular screening group had a university education than those in the irregular screening group. In the regular screening group, 56.12% of the women had an annual household income ranging from RMB 10,000 to 30,000. Notably, 2.94% and 0.86% of the women in the regular and irregular screening groups, respectively, received HPV immunization. A comprehensive summary of the participants’ characteristics is provided in Table 1.

**Table 1:** Characteristics of participants before and after matching in regular and irregular screening groups (n=2303)

Characteristics	Before		t/χ^2^	P	After		t/χ^2^	P
	Regular N=419	Irregular N=1884			Regular N=417	Irregular N=417		
Age	47.54±8.48	49.26±8.45	-3.77	＜0.001	47.65±8.35	47.65±8.35	0.00	1.00
HPV			0.0355	0.850			90.7092	＜0.001
Positive	86(20.53)	379(20.12)	-	-	84(20.14)	216(51.80)	-	-
Negatives	333(79.74)	1505(79.88)	-	-	333(79.86)	201(48.20)	-	-
Occupation			33.8342	＜0.001			6.1747	0.045
Farmers	165(39.76)	970(51.98)	-	-	165(39.95)	191(46.59)	-	-
Worker/Individual/Staff	169(40.72)	497(26.63)	-	-	168(40.68)	133(32.44)	-	-
Others	81(19.52)	399(21.38)	-	-	80(19.37)	86(20.98)	-	-
Education			31.5662	＜0.001			7.0053	0.030
Primary or illiterate	132(32.04)	832(44.40)	-	-	132(32.30)	166(39.90)	-	-
Middle/High school	195(47.33)	816(43.54)	-	-	195(47.56)	188(45.19)	-	-
Graduate	85(20.63)	226(12.06)	-	-	83(20.24)	62(14.90)	-	-
Annual income			82.9695	＜0.001			42.1386	＜0.001
≤10000	30(7.19)	300(15.98)	-	-	30(7.23)	50(12.80)	-	-
10000-30000	234(56.12)	629(33.51)	-	-	233(56.14)	141(34.06)	-	-
＞30000	135(32.37)	758(40.38)	-	-	134(32.29)	188(45.41)	-	-
Unknown	18(4.32)	190(10.12)	-	-	18(4.34)	35(8.45)	-	-
Family history of cancer			0.8549	0.3552			1.3287	0.2490
Yes	29(6.97)	156(8.34)	-	-	28(6.76)	37(8.92)	-	-
No	387(93.03)	1715(91.66)	-	-	386(93.24)	378(91.08)	-	-
Current gynecological symptoms			19.1545	＜0.001			30.0013	＜0.001
Yes	137(32.70)	836(44.37)	-	-	135(32.37)	213(51.08)	-	-
No	282(67.30)	1048(55.63)	-	-	282(67.63)	204(48.92)	-	-
Sex			2.0478	0.152			0.0147	0.903
Yes	256(85.91)	1461(82.54)	-	-	254(85.81)	354(86.13)	-	-
No	42(14.09)	309(17.46)	-	-	42(14.19)	57(13.87)	-	-
History of HPV vaccination			11.9414	0.0005			7.4675	0.0063
Yes	12(2.94)	16(0.86)	-	-	12(2.96)	2(0.48)	-	-
No	396(97.06)	1849(99.14)	-	-	394(97.04)	412(99.52)	-	-
Regular gynecological examinations			771.8798	＜0.001			347.5194	＜0.001
Yes	362(86.40)	331(17.57)	-	-	362(86.81)	94(22.54)	-	-
No	57(13.60)	1553(82.43)	-	-	55(13.19)	323(77.46)	-	-

Table 2 shows that age was a risk factor for HPV infection (odds ratio [OR]=1.056, 95% confidence interval [CI]: 1.031-1.082). In contrast, regular screening was a protective factor for HPV infection (OR=0.174, 95% CI: 0.117-0.259), suggesting that individuals who undergo regular screening have a lower likelihood of infection with HPV compared with those who do not.

**Table 2:** Multivariable logistic regression analysis of HPV infection

Variables	β	SE	Wald χ2	p	OR	95% CI
Age	0.054	0.012	19.787	＜0.001	1.056	[1.031-1.082]
Worker/Individual/Staff	-0.841	0.264	10.135	0.001	0.431	[0.257-0.724]
Other occupations	-0.908	0.264	11.791	0.001	0.403	[0.240-0.677]
Middle/High school	-0.441	0.220	4.016	0.045	0.643	[0.418-0.990]
Graduate	-0.674	0.363	3.435	0.064	0.510	[0.250-1.039]
Regular screening	-1.749	0.203	74.237	＜0.001	0.174	[0.117-0.259]
Known HPV	0.376	0.217	3.011	0.083	1.456	[0.952-2.226]

Table 3 shows that, among the individuals who had HPV knowledge (666), 216 (32.43%) underwent regular screening, whereas among the 1,637 participants who lacked HPV knowledge, 203 (12.40%) underwent regular screening (X2=127.6314, P<0.001).

**Table 3:** HPV awareness and regular screening among 2303 participants

Variable	Regular screened	Irregular screened	Total
Knowledge of HPV	216(32.43%)	450(67.57%)	666
No knowledge of HPV	203(12.40%)	1434(87.60%)	1637
Total	419(18.19%)	1884(81.81%)	2303

To enhance the comparability between the two groups, we paired them according to age, resulting in 417 matched pairs with an equal number of participants in both groups after matching(Table 1). After matching, the two groups were of equal age. Compared with the irregular screening group, the regular screening group was more educated and had a higher annual household income (P<0.05). There were 216 (51.80%) positive HPV test results in irregular screening and 84 (20.14%) in regular screening, (p<0.001). After matching, 165 (39.95%) and 191 (46.59%) women in the regular screening group and irregular screening group were farmers by occupation, respectively (p=0.045). Overall, the educational level of women in the matched regular screening group was higher than that of irregularly screened women. The number of regular gynecological check-ups was also higher in the regular plus cervical cancer screening group 362 (86.81%) than in the irregular screening group (p<0.001).

Table 4 shows the differences in HPV knowledge between the regular and irregular screening groups. Regarding the question “Do you know about HPV?” the number of individuals with HPV knowledge in the regular screening group (214 [51.32%]) was higher than that in the irregular screening group (107 [25.66%]) (P<0.0001). Among the participants with HPV knowledge, no significant difference was found between the regular and irregular screening groups in terms of HPV-related diseases and transmission, prognosis, and prevention of HPV infection.

**Table 4:** Knowledge of HPV between the regular screening group and the irregular screening group *The number of respondents who answered “YES” to the “Do you know about HPV?” question.

Questions	Answer	Regular	Irregular	χ^2^	P
		N(%)	N(%)		
Do you know about HPV?	Yes	214(51.32)	107(25.66)	57.9844	
	No	203(48.68)	310(74.34)		
HPV is an important etiological factor of cervical cancer*	Know	195(90.28)	99(92.52)	0.4415	0.506
	Unknown	21(9.72)	8(7.48)		
HPV can cause genital warts in both males and females*	Know	145(67.13)	67(63.81)	0.3473	0.555
	Unknown	71(32.87)	38(36.19)		
HPV can be transmitted through sexual contact*	Know	172(79.26)	84(78.50)	0.0248	0.874
	Unknown	45(20.74)	23(21.50)		
Most HPV infections can naturally regress without treatment*	Know	119(57.77)	48(48.00)	2.5905	0.107
	Unknown	87(42.23)	52(52.00)		
Men can also be infected with HPV*	Know	133(61.57)	60(56.60)	0.7315	0.392
	Unknown	83(38.43)	46(43.40)		
Condoms can prevent HPV infection*	Know	164(76.28)	82(77.36)	0.0462	0.829
	Unknown	51(23.72)	24(22.64)		

Table 5 shows attitudes toward the HPV vaccine among the regular screening group and the irregular screening group. In the regular screening group, 237 (56.83%) individuals were aware that “HPV infection can be prevented by vaccination,” whereas in the irregular screening group, 194 (46.52%) individuals had this knowledge (P=0.0029). Regarding “willingness to receive HPV vaccination,” most of the participants in both the regular (348 [83.45%]) and irregular (345 [82.73%]) screening groups demonstrated a positive attitude toward HPV. Regarding the willingness to have their daughters vaccinated against HPV, mothers in the irregular screening group (17 [6.97%]) exhibited a higher level of hesitancy compared with mothers in the regular screening group (4 [1.70%], P=0.004).

**Table 5:** Attitudes toward HPV vaccine among the regular screening group and the irregular screening group

Questions	Answer	Regular	Irregular	χ^2^	P
		N (%)	N (%)		
HPV infection can be prevented by vaccination	-	-	-	8.8781	0.0029
-	Yes	237(56.83)	194(46.52)	-	-
-	No	180(43.17)	223(53.48)	-	-
Willingness to receive HPV vaccination	-	-	-	0.0768	0.781
-	Yes	348(83.45)	345(82.73)	-	-
-	No	69(16.55)	72(17.27)	-	-
Whether there are daughters	-	-	-	0.0171	0.896
-	Yes	246(60.59)	249(60.14)	-	-
-	No	160(39.41)	165(39.86)	-	-
Willingness to allow your daughter to receive HPV vaccination	-	-	-	7.9162	0.004
-	Yes	231(98.30)	227(93.03)	-	-
-	No	4(1.70)	17(6.97)	-	-

**Figure 1:**
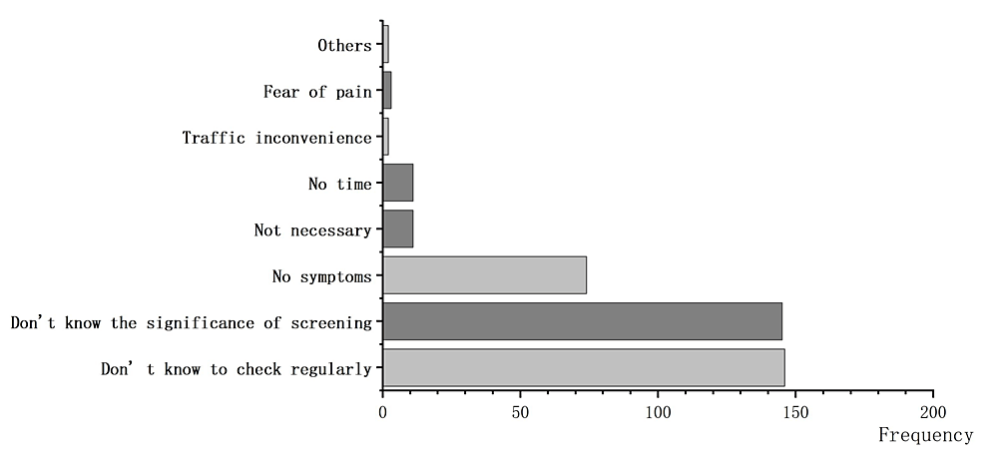
Reasons for not having regular screening

A total of 295 women provided reasons for not undergoing regular screening. Multiple reasons could be simultaneously selected. “Do not know how to check regularly” was the most frequently selected reason, followed by “Do not know the significance of screening.” The third reason was “No symptoms,” and a small percentage of reasons included feeling unnecessary and having no time. Figure 1 shows the reasons for not having regular screening.

## Discussion

This study mainly comprised women in underdeveloped areas, with the objective of assessing the patterns of regular screening behavior and the level of awareness regarding cervical cancer among this specific demographic. Our study demonstrated that women who were regularly screened were less likely to be infected with HPV than those who were irregularly screened, which suggests the importance of regular screening. Moreover, awareness of cervical cancer was higher in the regular screening group than in the irregular screening group. These results provide a basis for the development of grassroots public health work emphasizing the importance of cervical cancer screening and health education. This is particularly crucial in low-income areas where targeted efforts can significantly contribute to improving healthcare outcomes.

In our study, the percentage of women screened in the past 3 years was 18.01%, which is markedly lower than the regular screening rate among women in urban areas (82.14%), according to a previous study [14]. In 2009, China launched the National Cervical Cancer Screening Program for Rural Areas, providing complimentary cervical cancer screening to registered rural women aged 35-64 years. Over the last 10 years, the rates of cervical cancer screening have risen in both urban and rural regions of China [15]. However, these rates still fall below the WHO target of achieving 70% coverage for cervical cancer screening. Furthermore, disparities persist, revealing inequalities in screening rates between urban and rural areas and between the eastern and western regions of the country [13]. Factors such as inadequate or low income, elevated costs associated with attending screening (including transportation constraints and time commitments), and the absence of free employee check-ups, can contribute to reduced willingness to undergo screening for cervical cancer [5]. Screening rates tend to be lower in older than in younger women. Specifically, women aged >50 years exhibit significantly lower screening rates than those in the 35-49-year age group, as indicated in a previous study [16]. Our own study corroborates these findings, revealing that women in the regular screening group were generally younger than those in the irregular screening group. This phenomenon can be attributed to lower awareness of cervical cancer and entrenched traditional beliefs among older women [17].

Another significant factor contributing to low rates of cervical cancer screening is the limited awareness of screening, which poses a common barrier, especially in low- and middle-income countries. Several studies have consistently shown a positive correlation between cognitive awareness and the willingness to participate in screening [18,19]. This aligns with findings from cervical cancer awareness surveys conducted in various regions [20,21]. In line with these observations, our study also indicates that women in this economically disadvantaged area possess low knowledge about cervical cancer. A common belief among women is that screening is unnecessary if they are not experiencing symptoms [22]. This lack of awareness regarding the early features and risk factors for cervical cancer can result in patients with early stage cervical cancer missing an optimal window for treatment. Therefore, it is crucial to enhance awareness among women to promote screening and ensure timely intervention.

Health education is an effective method for raising awareness about cervical cancer. Providing education to women through lectures and video dissemination results in a substantial increase in their willingness to undergo cervical cancer screening. In Abera’s study, the intervention group demonstrated a remarkable and sustained increase of 46.4% in screening willingness during subsequent follow-up visits. This underscores the importance of health education in fostering women’s participation in screening initiatives [23-25]. Nevertheless, notably, women in underdeveloped areas, particularly older women, may not have received systematic education, face challenges in reading and writing, or may have limited access to useful information via mobile phones. Additionally, owing to their geographical distance, they may encounter difficulties in accessing public health services. Undertaking health education initiatives for this demographic poses a significant challenge given these factors. Addressing these challenges requires innovative and targeted approaches to ensure the effective communication and education of women in underdeveloped areas.

HPV vaccination is considered the primary preventive measure against cervical cancer [26]. Observations in Italy and Norway among women who have received the vaccine indicate a decrease in HPV disease incidence rates, underscoring the necessity and effectiveness of the vaccine [27,28]. However, in China, only a few cities with a high socioeconomic status have implemented free HPV vaccination programs [29]. In our study, although women who were not screened regularly exhibited lower socioeconomic status and awareness of HPV compared to those who underwent regular screenings, they still demonstrated a comparable willingness to accept HPV vaccination. This may be attributed to the high costs associated with HPV vaccination, difficulties in scheduling appointments, and a lack of supportive policies, which hindered their uptake [30]. The Lueyang County government recently devised a plan to implement a flat-rate subsidy for the cost of preventive HPV vaccination for girls aged 9-14 years within the county. This initiative aims to encourage HPV vaccination among girls of appropriate age and increase the local vaccination rate. However, notably, the vaccine has a limited timeframe and does not encompass all HPV strains. Even with vaccination, regular cervical cancer screening is essential for comprehensive prevention. The integration of vaccination and screening is crucial for the effective implementation of primary and secondary prevention measures against cervical cancer.

Limitations: This study has some limitations. First, our study specifically targeted women in less-developed regions to provide evidence of the importance of regular screening in economically disadvantaged areas. It is not intended for extrapolation to regions with higher economic levels and does not claim national applicability. Second, this was a cross-sectional study, and prospective observational studies are required to obtain long-term outcomes in regularly and irregularly screened women. Third, although we identified an association between screening behavior and awareness, a causal relationship was not established. This underscores the need for further studies to provide evidence-based insights into subsequent cervical cancer prevention and management in Lueyang County. Continued investigation will enhance our understanding of the factors influencing screening behavior and aid in the development of more targeted and effective preventive strategies.

## Conclusions

The proportion of women in Lueyang County who undergo regular cervical cancer screening is less than 50%. Women who undergo regular screening have a better understanding of HPV and HPV vaccines than those who do not undergo regular screening. The government should provide extensive free screening services, especially in economically disadvantaged areas. Moreover, it is crucial to introduce targeted and efficient public health education initiatives aimed at bolstering women's understanding of HPV and cervical cancer, along with emphasizing the significance of regular cervical cancer screening.

## Appendices

Summary of visits recorded

*1. First consultation time

*2. I.D. number

*3. HPV diagnosis

 negatives    positive

*4. HPV typing

 6

 11

 40

 42

 43

 44

 54

 61

 70

 72

 81

 cp6 108

 16

 18

 31

 33

 35

 39

 45

 51

 52

 56

 58

 59

 68

 73

 82

 66

 26

 53

 66

 89

 83

 69

*5. Need for follow-up

 Yes    No

Patient basic information

Basic Information

*1.Your birthdate is

*2.Date of consultation

*3. Access to medical services

 Voluntariness

 Advice from  relatives or friends

 Advice from a doctor

 Other reasons

4. Your Employment Status

 Unemployed

 Part-time employment

 Full-time employment

 Students enrolled

 Farmers work the land

 Retirement (from work)

 Others

 Unknown

5. Your Occupation

 Farmer

 Self-employed

 Employee of an institution

 Workers

 Others

Please specify other occupations.

6. Your level of education

 Illiteracy/no formal education

 Primary and below

 Junior high school

 Senior high school

 Junior college

 Undergraduate

 Master's degree

 Doctor's degree

 Unknown

7. Your place of origin

8. Your Nationality

 Han    Qiang    Other

Please fill in other nationalities

9.Your Religious Beliefs

 No

 Buddhism

 Christianity

10.Number of family members

11.Annual personal income(¥,yuan)

 <3 thousands

 3-5 thousands

 5 thousands-10 thousands

 10-30 thousands

 30-100 thousands

 >100 thousands

 Unwilling to disclose

 Unknown

12. Annual household income(¥,yuan)

 <5 thousands

 5 thousands-10 thousands

 10-30 thousands

 30-100 thousands

 >100 thousands

 Unwilling to disclose

 Unknown

13. Whether enrolled in medical insurance

 Yes    No

13.1 Type of participating health insurance

13.2 Other types of health insurance

14. Living environment and living conditions

15. Smoking status

 Never    Quit smoking    Not having quit smoking

How many cigarettes per day

16. Alcohol consumption

 Never    Quit drinking    Not having quit drinking

Current medical history

1. Increased vaginal discharge

 Yes    No

2. Bleeding from sexual intercourse

 Yes    No

3. Low back pain or lower back and abdominal soreness

 Yes    No

4. Frequent, urgent, painful urination

 Yes    No

5. Other symptoms

 Yes    No

Past medical history

1. Gynecological history

 Pelvic inflammation

 Adenomyosis

 Endometriosis

 History of endarterectomy (ablation)

 History of gestational trophoblastic disease

 History of tubal anomalies

 History of ectopic pregnancy

 History of ovarian abnormalities

 Carcinoma

 Diseases of the lower genital tract

 Other medical history

2. Previous surgeries

 Yes    No

3. Are there any traumatic injuries

 Yes    No

4. History of blood transfusion and donation

 Yes    No

5. Allergy history

 Yes    No

6. Regular screening

 Yes    No

6.1TCT

 Yes    No

Inspection frequency

6.2HPV

 Yes    No

Inspection frequency

7. Whether or not you have been vaccinated against HPV

 Yes    No

7.1 Vaccine type

 bivalent vaccine    quadrivalent vaccine    nine-valent vaccine

7.2 Vaccination schedule

8. Ever been diagnosed with warts

 Yes    No    Unknown

9. Ever been diagnosed with cervicitis

 Yes    No    Unknown

10. Ever been diagnosed with a sexually transmitted disease

 Yes    No    Unknown

11. Bleeding during previous sexual activity

 Yes    No    Unknown

12. Any other areas of pain

 Yes    No 

Menses history

1. Time of menarche

2. Date of last menstrual period

3. Menopausal

 Yes    No 

3.1 age at menopause

Marital and reproductive history

1. Age of marriage (years old)

2. Age of sexual debut (years old)

3. Age at first delivery (years old)

4. Number of pregnancies

 0

 1

 2

 3

 4

 5

 6

 7

 >=8

5. Number of deliveries

6. Mode of delivery

caesarean section    natural birth (without surgical operation) 

7. Place of delivery

 Home    Hospitals    Both

8. Number of marriages

Family history of inheritance

1. Have a family history of tumors

 Yes    No 

1.1 Tumor site

1.2 Patient-Self Relationship

hygiene situation

1. Number of sexual partners

2. Whether or not there is a requirement to have children

 Yes    No 

3. Have a sex life?

 Yes    No

3.1 Sexual frequency

 ≤1 time/week

 2 times/week

 3 times/week

 ≥4 times/week

 1 time/2 weeks

 1 time/3 weeks

 ≥1 time/4 weeks

4.Willingness to use contraception

 Yes    No 

4.1 Method of contraception

 Intrauterine device (IUD)

 Extracorporeal contraception

 Oral contraceptive pill (OCP)

 Condoms

 Tubal ligation

 Uncontraceptive

 Menopausal

 Other

5. Attitudes towards condoms

 Like

 Acceptable

 Dislike

6. Wash the vulva before intercourse

 Yes

 No

 Sometimes

7. Wash your hands before washing your vulva

 Yes

 No

 Sometimes

8. Wash your vagina before intercourse

 Yes

 No

 Sometimes

9. Washing the vulva after intercourse

 Yes

 No

 Sometimes

10. Vaginal washing after intercourse

 Yes

 No

 Sometimes

11. Showering after intercourse

 Yes

 No

 Sometimes

Awareness of HPV

1. Do you know about HPV (If you choose “yes”, please continue with the questions that follow)

 Yes

 No

2. HPV is an important etiological factor of cervical cancer

 Know   Unknown

3. HPV can cause genital warts in both males and females

 Know   Unknown

4. HPV can be transmitted through sexual contact

 Know   Unknown

5. Most HPV infections can naturally regress without treatment

 Know   Unknown

6. Men can also infection HPV

 Know   Unknown

7. Condoms can prevent HPV infection

 Know   Unknown

Awareness of regular screening

1. Whether regular gynecological checkups are done (Including cervical smears）

 Yes    No 

2. Reasons for previous gynecological examination

 Symptomatic

 Routine Physical Examinations

 Thinking that gynecological exams can solve all gynecological problems

 Medical recommendation

 Free medical checkup program

 Other

3. Regular cervical cancer screening

 Yes    No

4. Reasons for not having regular cervical cancer screening

 Don' t know to check regularly

 Don't know the significance of screening

 No symptoms

 Not necessary

 No time

 No money

 Traffic inconvenience

 Embarrass

 Fear of pain

 Other

Attitudes toward HPV vaccine

1. HPV infection can be prevented by vaccination

 Known  Unknown

2. Willingness to receive HPV vaccination

 Yes    No 

2.1. Reasons you don't want to get the HPV vaccine

 There are no risk for cervical cancer

 It doesn't make any difference if you're inoculated or not.

 The HPV vaccine is not yet widely available

 Inoculation can have varying degrees of side effects

 Distrust in the source of HPV vaccine

 Unable to afford vaccination

2.2. Reasons why you would like to receive the HPV vaccine

 Considered to be of benefit

 You can get cervical cancer if you don't get vaccinated

 You can get HPV if you don't get vaccinated

 You can get genital warts if you don't get inoculated.

 Fear that you have been infected with HPV

3. Do you have a daughter

 Yes    No 

3.1 If you have a daughter would you want your daughter to get the HPV vaccine?

 Yes    No 

3.2 Reasons why you don't want your daughter to get the HPV vaccine

 Doubts about the safety and efficacy of vaccines

 Mistrust of the source of HPV

 Worried about not being able to afford the cost

 Thinks daughter is too young to be vaccinated

 Other

Your daughter's age

Other reasons

4. You want the HPV vaccine to prevent

 Cervix cancer

 Genital warts

 Both

 Other

5. Price of HPV vaccine you can afford (per vaccination)

 <50 yuan

 50-100 yuan

 100-200 yuan

 200-500 yuan

 >=500 yuan

6. What would make you more likely to want to get the HPV vaccine?

 Doctor or nurse recommendation

 Recommendations from family members or friends

 Medical Health Education

 Health education in schools

 Media Outreach

7. What is your preferred payment route for HPV vaccination?

 Self-financed vaccination

 Insurance company pays all costs

 Insurance companies cover part of the cost

 Full cost to the Government

 Full part of the cost to the Government

8.Your preferred age range for the HPV vaccine
