# Correction: Knowledge and Practices Regarding Human Papillomavirus and Cervical Cancer Screening Among Women in Low-Income Areas of China: A Cross-Sectional Study

**DOI:** 10.7759/cureus.c174

**Published:** 2024-05-10

**Authors:** Jiaojiao Chen, Ruoyi Zhang, Wei Xu, Li Bai, Dehua Hu, Yuxian Nie, Rumei Xiang, Dan Kang, Qiu-ling Shi

**Affiliations:** 1 College of Public Health, Chongqing Medical University, Chongqing, CHN; 2 Obstetrics and Gynecology, Centre of Maternal and Child Health, Shaanxi, CHN; 3 State Key Laboratory of Ultrasound in Medicine and Engineering, Chongqing Medical University, Chongqing, CHN; 4 College of Public Health, State Key Laboratory of Ultrasound in Medicine and Engineering, Chongqing Medical University, Chongqing, CHN

This article has been corrected to accurately reflect the affiliation of the first author. 

Jiaojiao Chen’s affiliation has been changed from School of Public Health, Chongqing Medical University, Chongqing, CHN to College of Public Health, Chongqing Medical University, Chongqing, CHN.

In the original version, disclosures about the funding for this project were omitted. Funding for this project was provided by:

Foundation of State Key Laboratory of Ultrasound in Medicine and Engineering (No. 2021KFKT007) Chongqing Social Science Planning Project (No. 2022BS081) China Postdoctoral Science Foundation Funded Project (No. 2022MD723732) Natural Science Foundation of Chongqing, China (No. 2023NSCQ-BHX0036)

The authors deeply regret that these errors were not identified and addressed prior to publication.

